# Supplementary figures and images for: Dynamics of allosteric regulation of the phospholipase C-γ isozymes upon recruitment to membranes
Source: eLife. 2022 Jun 16;11:e77809. doi: 10.7554/eLife.77809 (PMC9203054; doi:10.7554/eLife.77809)

Figure 1-figure supplement 1-source data 1

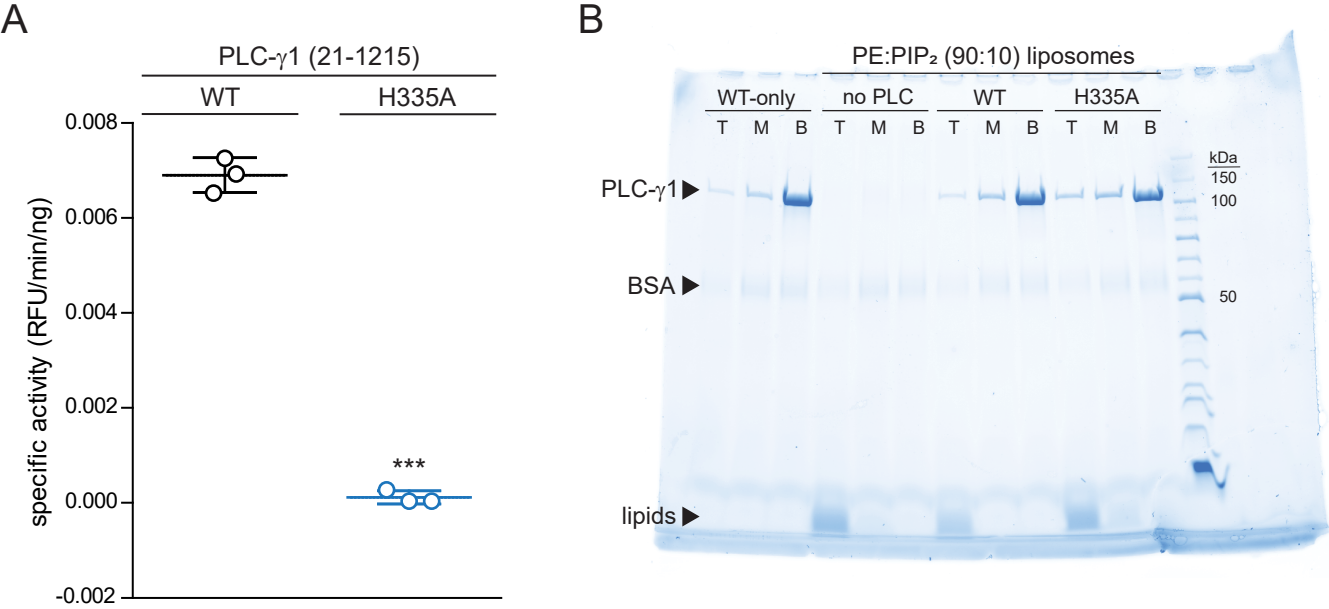

Supplement: Figure 1—source data 2. — Raw images of gels used to produce final figures. [file elife-77809-fig1-data2.zip › Figure 1-figure supplement 1-source data 1.pdf]

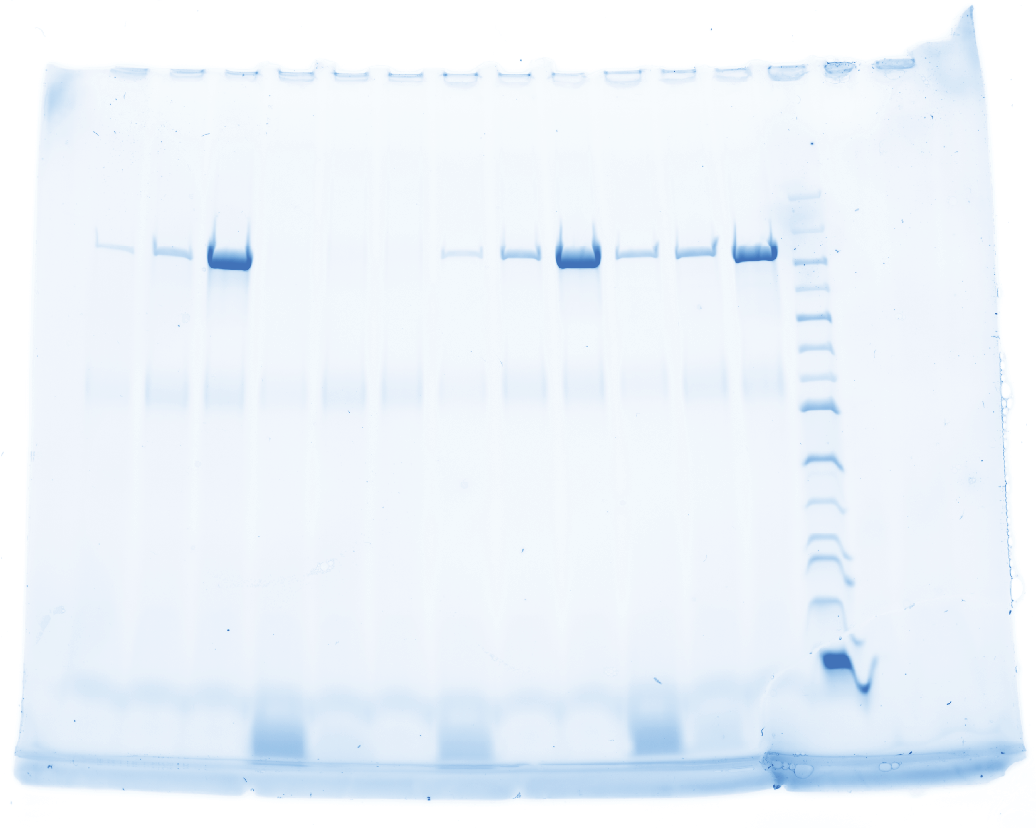

Supplement: Figure 1—source data 2. — Raw images of gels used to produce final figures. [file elife-77809-fig1-data2.zip › Figure 1-figure supplement 1-source data 2.png]

Figure 2-figure supplement 1-source data 1

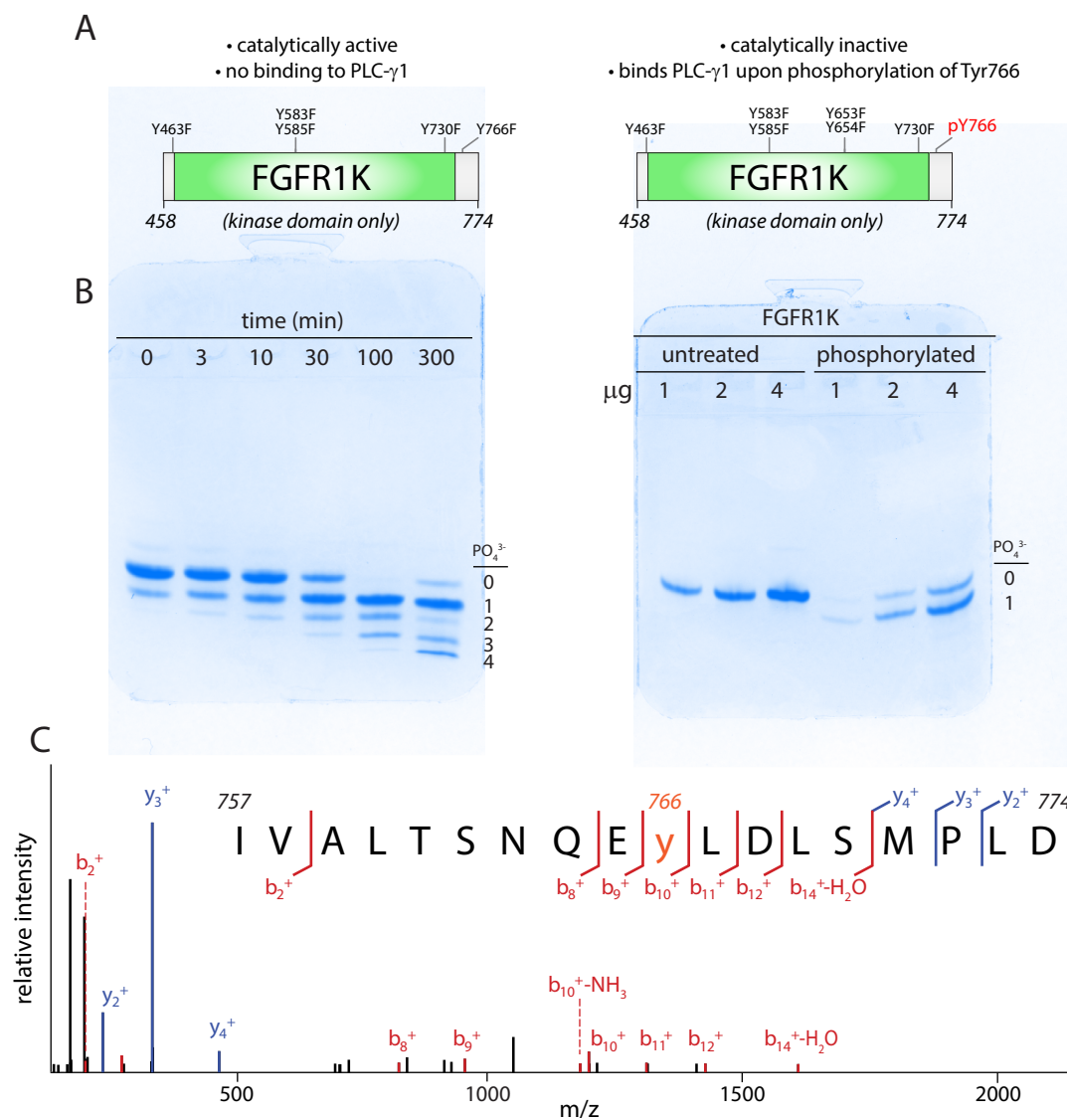

Supplement: Figure 1—source data 2. — Raw images of gels used to produce final figures. [file elife-77809-fig1-data2.zip › Figure 2-figure supplement 1-source data 1.pdf]

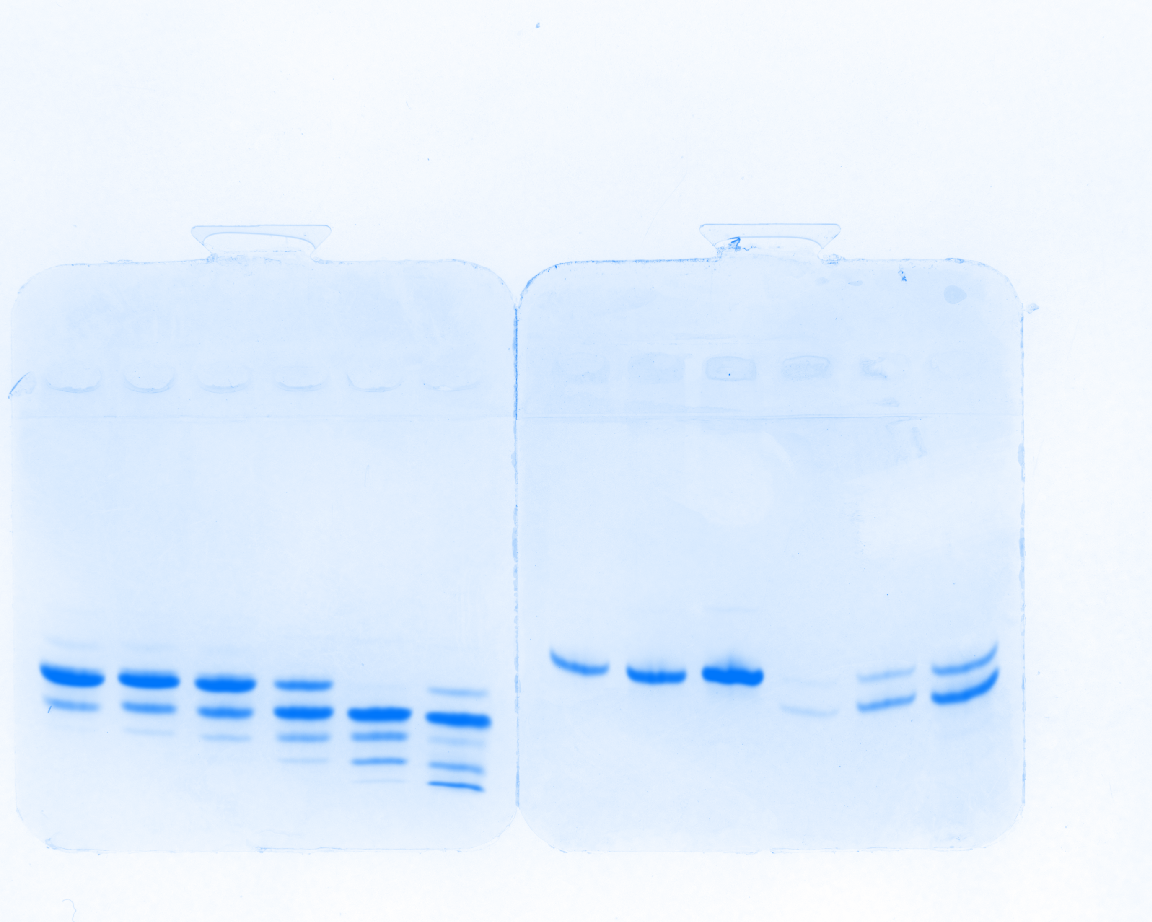

Supplement: Figure 1—source data 2. — Raw images of gels used to produce final figures. [file elife-77809-fig1-data2.zip › Figure 2-figure supplement 1-source data 2.png]

Figure 2-figure supplement 2-source data 1

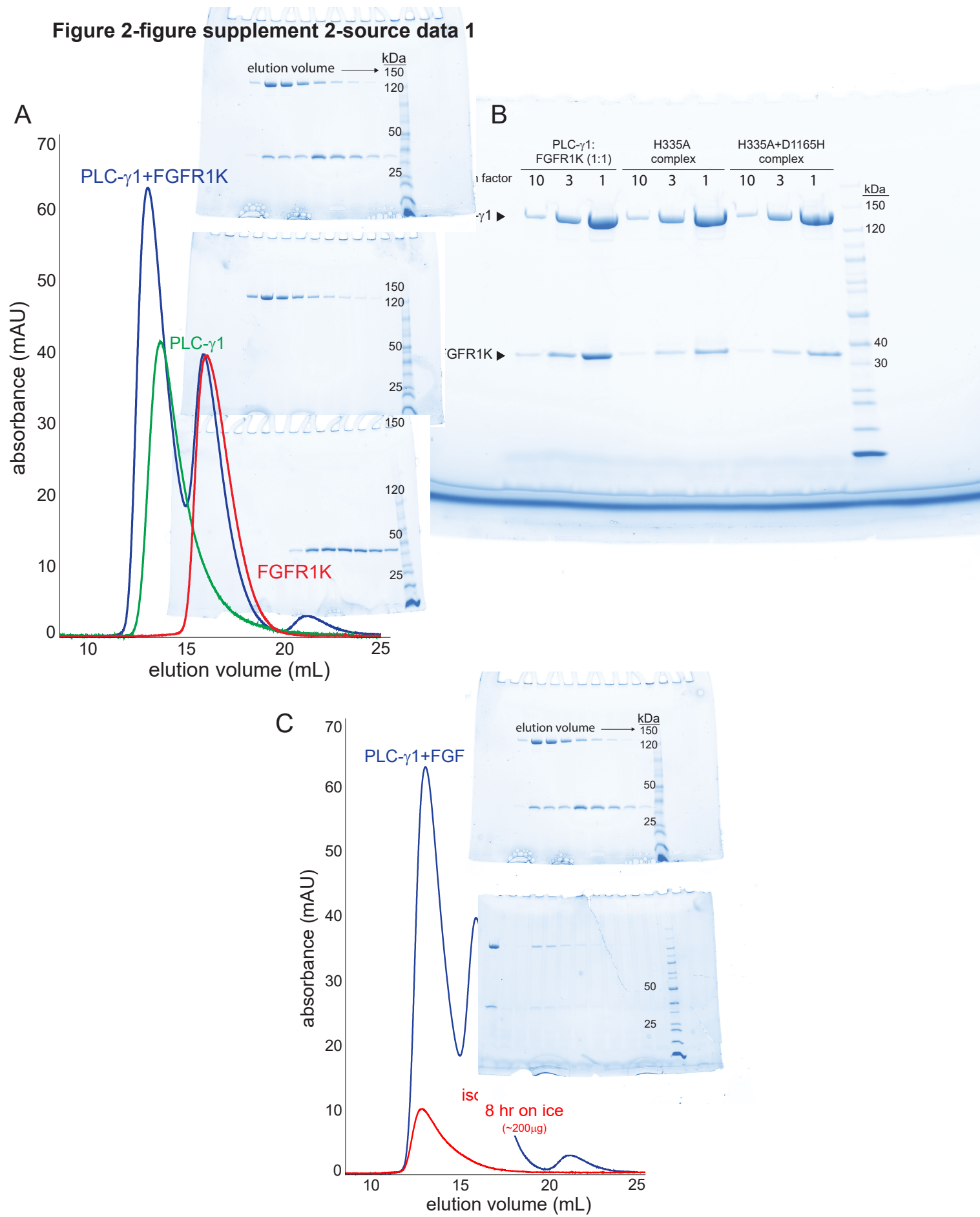

Supplement: Figure 1—source data 2. — Raw images of gels used to produce final figures. [file elife-77809-fig1-data2.zip › Figure 2-figure supplement 2-source data 1.pdf]

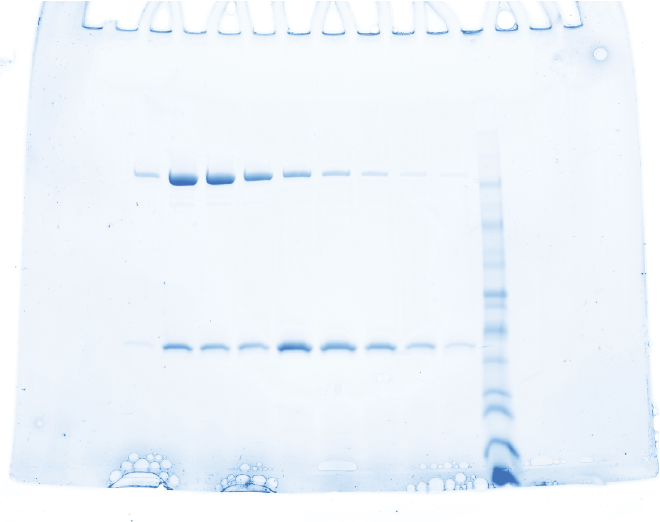

Supplement: Figure 1—source data 2. — Raw images of gels used to produce final figures. [file elife-77809-fig1-data2.zip › Figure 2-figure supplement 2-source data 2.png]

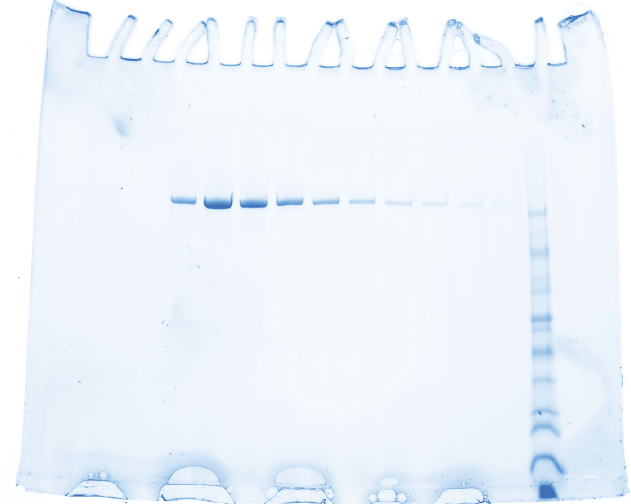

Supplement: Figure 1—source data 2. — Raw images of gels used to produce final figures. [file elife-77809-fig1-data2.zip › Figure 2-figure supplement 2-source data 3.png]

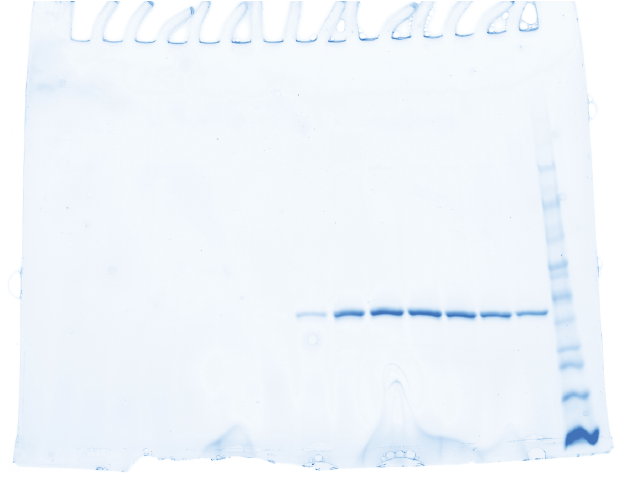

Supplement: Figure 1—source data 2. — Raw images of gels used to produce final figures. [file elife-77809-fig1-data2.zip › Figure 2-figure supplement 2-source data 4.png]

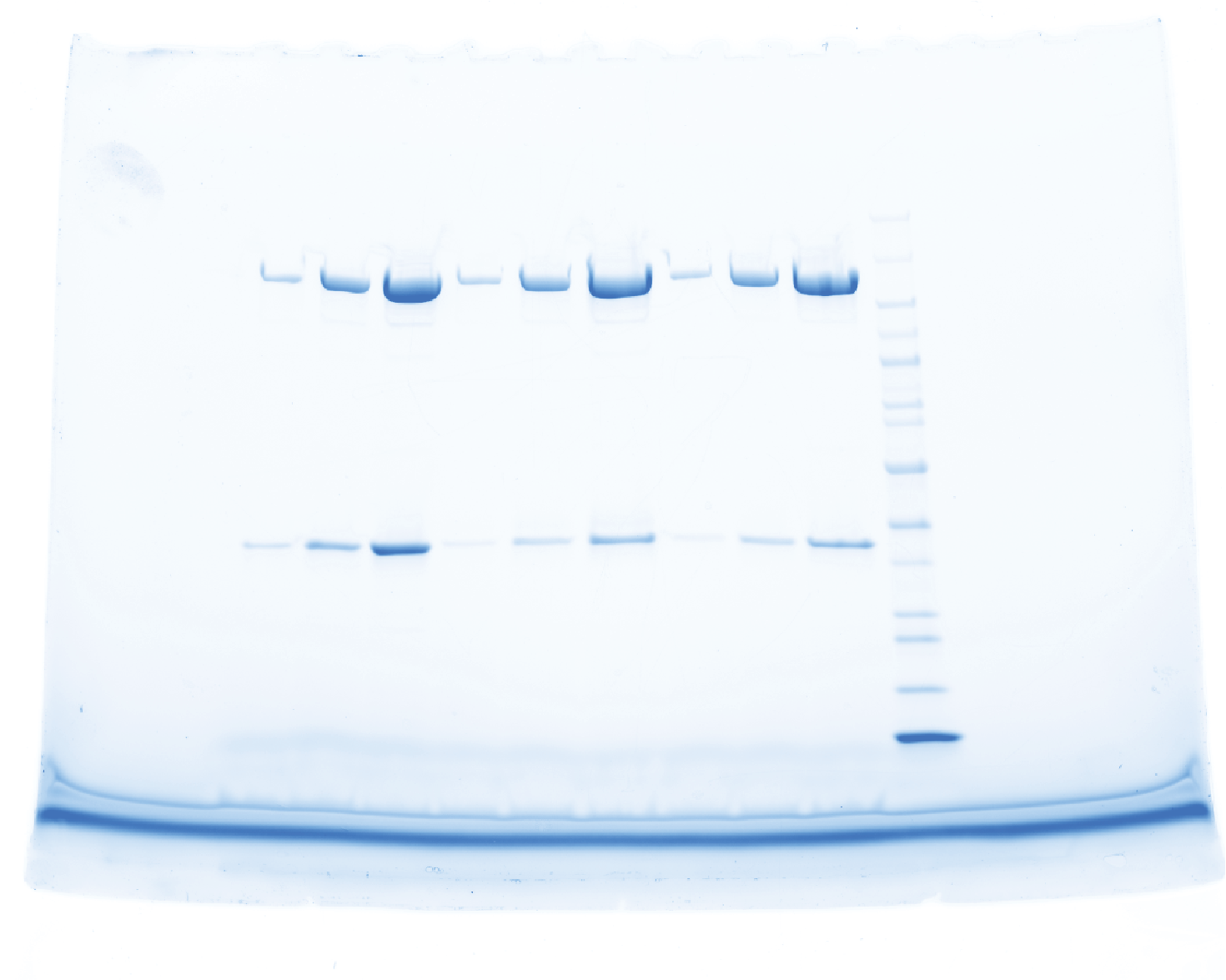

Supplement: Figure 1—source data 2. — Raw images of gels used to produce final figures. [file elife-77809-fig1-data2.zip › Figure 2-figure supplement 2-source data 5.png]

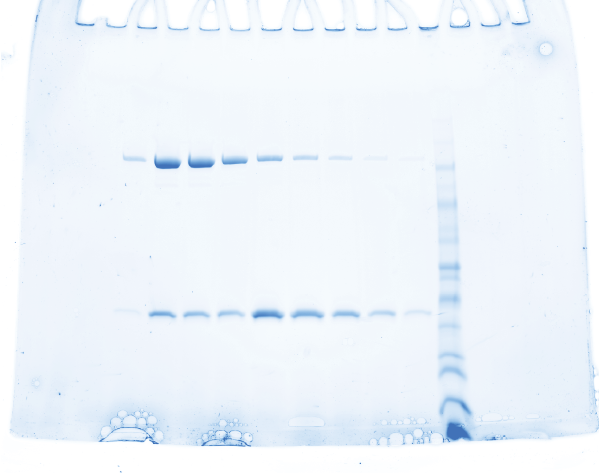

Supplement: Figure 1—source data 2. — Raw images of gels used to produce final figures. [file elife-77809-fig1-data2.zip › Figure 2-figure supplement 2-source data 6 (same as Figure 2-figure supplement 2-source data 2).png]

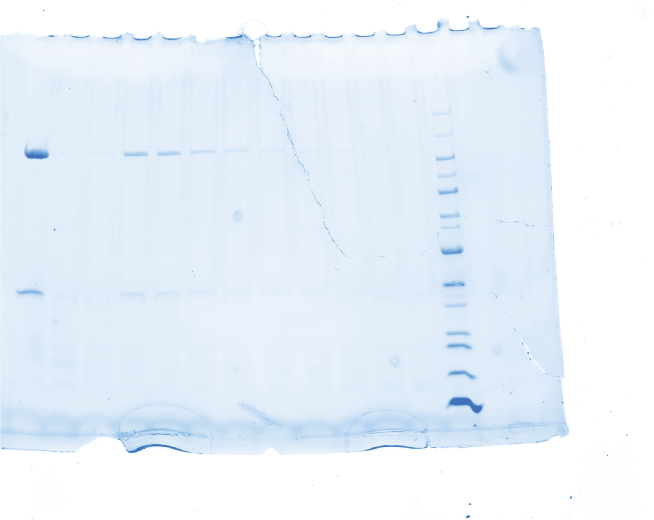

Supplement: Figure 1—source data 2. — Raw images of gels used to produce final figures. [file elife-77809-fig1-data2.zip › Figure 2-figure supplement 2-source data 7.png]
