# Supplementary material for: Dynamics of allosteric regulation of the phospholipase C-γ isozymes upon recruitment to membranes
Source: eLife. 2022 Jun 16;11:e77809. doi: 10.7554/eLife.77809 (PMC9203054; doi:10.7554/eLife.77809)
Supplement: Supplementary file 1. [file elife-77809-supp1.docx]

| **Data Set** | **PLCγ-1(CD)** | **PLCγ-1(CD) + FGFR** | **PLCγ-1(CD) + Lipid** | **PLCγ-1(CD) + FGFR + Lipid** |
| --- | --- | --- | --- | --- |
| HDX reaction details | %D_2_O=72%  pH_(read)_= 7.0  Temp= 18°C | %D_2_O=72%  pH_(read)_= 7.0  Temp= 18°C | %D_2_O=72%  pH_(read)_= 7.0  Temp= 18°C | %D_2_O=72%  pH_(read)_= 7.0  Temp= 18°C |
| HDX time course | 3s, 30s, 300s, 3000s, 10000s | 3s, 30s, 300s, 3000s, 10000s | 3s, 30s, 300s, 3000s, 10000s | 3s, 30s, 300s, 3000s, 10000s |
| HDX controls | N/A | N/A | N/A | N/A |
| Back-exchange | Corrected based on %D_2_O | Corrected based on %D_2_O | Corrected based on %D_2_O | Corrected based on %D_2_O |
| Number of peptides | 254 | 254 | 254 | 254 |
| Sequence coverage | 91.5% | 91.5% | 91.5% | 91.5% |
| Average peptide length/ redundancy | Length = 13.0  Redundancy = 2.7 | Length = 13.0  Redundancy = 2.7 | Length = 13.0  Redundancy = 2.7 | Length = 13.0  Redundancy = 2.7 |
| Replicates | 3 | 3 | 3 | 3 |
| Repeatability | Average StDev = 0.4% | Average StDev = 0.4% | Average StDev = 0.5% | Average StDev = 0.5% |
| Significant differences in HDX | >5% and >0.4 Da and unpaired t-test <0.01 | >5% and >0.4 Da and unpaired t-test <0.01 | >5% and >0.4 Da and unpaired t-test <0.01 | >5% and >0.4 Da and unpaired t-test <0.01 |

| **Data Set** | **PLCγ-1(D1165)** | **PLCγ-1(D1165) + FGFR** | **PLCγ-1(D1165) + Lipid** | **PLCγ-1(D1165) + FGFR + Lipid** |
| --- | --- | --- | --- | --- |
| HDX reaction details | %D_2_O=72%  pH_(read)_= 7.0  Temp= 18°C | %D_2_O=72%  pH_(read)_= 7.0  Temp= 18°C | %D_2_O=72%  pH_(read)_= 7.0  Temp= 18°C | %D_2_O=72%  pH_(read)_= 7.0  Temp= 18°C |
| HDX time course | 3s, 30s, 300s, 3000s, 10000s | 3s, 30s, 300s, 3000s, 10000s | 3s, 30s, 300s, 3000s, 10000s | 3s, 30s, 300s, 3000s, 10000s |
| HDX controls | N/A | N/A | N/A | N/A |
| Back-exchange | Corrected based on %D_2_O | Corrected based on %D_2_O | Corrected based on %D_2_O | Corrected based on %D_2_O |
| Number of peptides | 254 | 254 | 254 | 254 |
| Sequence coverage | 91.5% | 91.5% | 91.5% | 91.5% |
| Average peptide length/ redundancy | Length = 13.0  Redundancy = 2.7 | Length = 13.0  Redundancy = 2.7 | Length = 13.0  Redundancy = 2.7 | Length = 13.0  Redundancy = 2.7 |
| Replicates | 3 | 3 | 3 | 3 |
| Repeatability | Average StDev = 0.4% | Average StDev = 0.4% | Average StDev = 0.4% | Average StDev = 0.4% |
| Significant differences in HDX | >5% and >0.4 Da and unpaired t-test <0.01 | >5% and >0.4 Da and unpaired t-test <0.01 | >5% and >0.4 Da and unpaired t-test <0.01 | >5% and >0.4 Da and unpaired t-test <0.01 |
